# Pointing the trident in the right direction: recognizing spinal neurosarcoidosis through a specific MRI pattern

**DOI:** 10.1055/s-0045-1807720

**Published:** 2025-05-13

**Authors:** Alena Khalil, Kevin J. Abrams, Márcio Luís Duarte, Leonardo Furtado Freitas

**Affiliations:** 1 Nova Southeastern University, Dr. Kiran C. Patel College of Osteopathic Medicine, Fort Lauderdale FL, United States.; 2 Baptist Health South Florida, Department of Radiology, Division of Clinical Neuroradiology, Miami FL, United States.; 3 Universidade de Ribeirão Preto, Departamento de Radiologia, Guarujá SP, Brazil.; 4 Diagnósticos da América S.A., São Paulo SP, Brazil.

**Figure 1:**
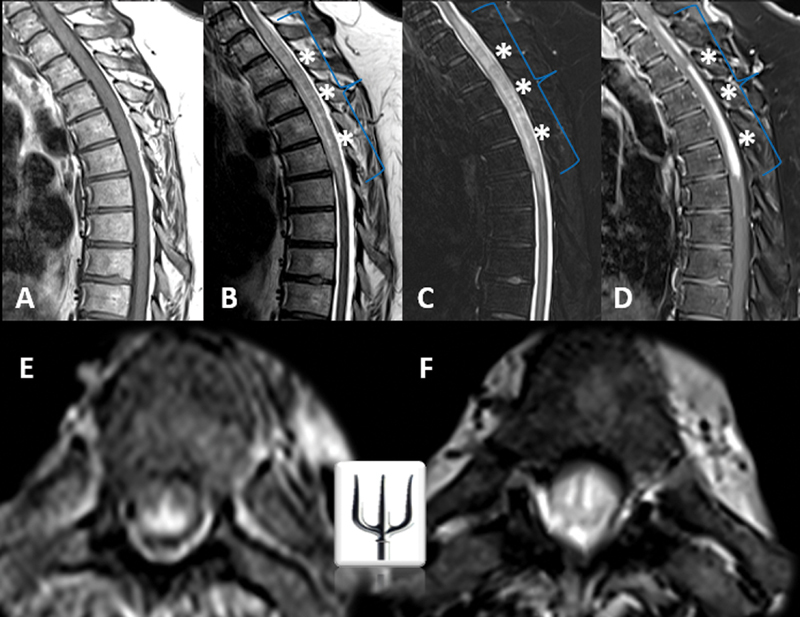
Magnetic resonance imaging of the thoracic spine in sagittal ( **A–D** ) and axial views ( **E–F** ). Evidence of longitudinally extensive transverse myelitis at the cervicothoracic junction is observed, associated with significant edema and spinal cord swelling (blue braces). Notably, a contrast-enhancing component (white asterisks) in the posterior column and central regions, extending toward the pial/subpial surface and central ependymal canal, demonstrates T2-weighted/ short tau inversion recovery (STIR) hyposignal and exhibits a “trident-shaped head” appearance on axial images. The dorsal enhancement likely results from granulomatous inflammation spreading via perivascular pathways and may be influenced by meningeal lymphatic drainage and increased vascular permeability, predisposing this region to inflammation.

**Figure 2:**
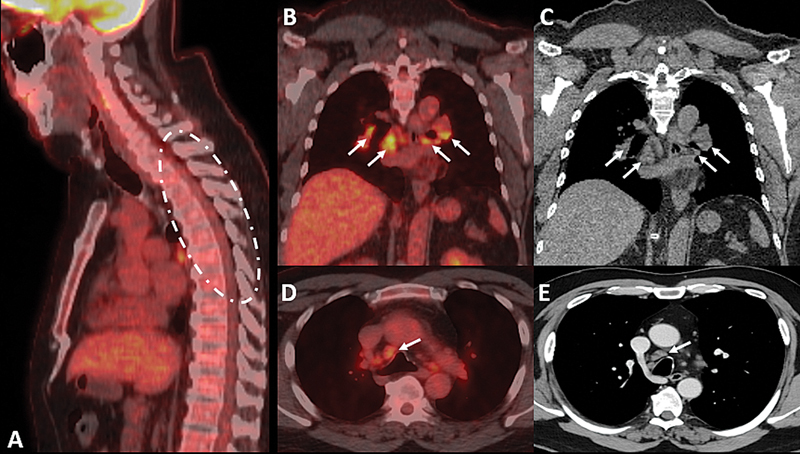
Positron emission tomography-computed tomography scan with fluorodeoxyglucose (FDG) in sagittal ( **A** ), coronal ( **B–C** ), and axial ( **D–E** ) views. No significant FDG uptake was observed in the cervicothoracic spinal cord lesion. However, multiple mediastinal, hilar, and precarinal lymphadenopathies demonstrated high radiotracer uptake, suggesting a granulomatous inflammatory process.

We herein report the case of a 51-year-old male presenting with progressive neurological symptoms, including numbness, tingling below the umbilicus, urinary difficulty, constipation, and weakness. Following a recent coronavirus disease 2019 (COVID-19) vaccination, magnetic resonance imaging (MRI) ( Figure 1 ) revealed longitudinally extensive transverse myelitis (LETM) with a trident-shaped pattern on axial sequences, a hallmark of spinal neurosarcoidosis. 1 2 3 Positron emission tomography-computed tomography ( Figure 2 ) demonstrated multiple hypermetabolic hilar and mediastinal lymphadenopathies, further supporting this diagnosis, particularly given the possibility of false-positive aquaporin-4-immunoglobulin G (AQP4-IgG) enzyme-linked immunosorbent assay (ELISA) results. 4 Early recognition of the trident sign enabled the prompt initiation of corticosteroids and immunosuppressive therapy, highlighting the diagnostic utility of this specific MRI pattern in an appropriate clinical scenario, with primary spinal cord lymphoma as a differential diagnosis. 5
